# Biological and Proteolytic Variation in the Venom of *Crotalus scutulatus scutulatus* from Mexico

**DOI:** 10.3390/toxins10010035

**Published:** 2018-01-08

**Authors:** Miguel Borja, Edgar Neri-Castro, Gamaliel Castañeda-Gaytán, Jason L. Strickland, Christopher L. Parkinson, Juan Castañeda-Gaytán, Roberto Ponce-López, Bruno Lomonte, Alejandro Olvera-Rodríguez, Alejandro Alagón, Rebeca Pérez-Morales

**Affiliations:** 1 Facultad de Ciencias Biológicas, Universdad Juárez del Estado de Durango, Av. Universidad s/n. Fracc. Filadelfia, C.P. 35010 Gómez Palacio, Dgo., Mexico; alessandro_53@hotmail.com (M.B.); gamaliel.cg@gmail.com (G.C.-G.); jjcg00@gmail.com (J.C.-G.); 2 Facultad de Ciencias Químicas, Universidad Juárez del Estado de Durango, Av. Artículo 123 s/n. Fracc. Filadelfia, Apartado Postal No. 51, C.P. 35010 Gómez Palacio, Dgo., Mexico; 3 Instituto de Biotecnología, Universidad Nacional Autónoma de Mexico, Avenida Universidad 2001, Chamilpa, C.P. 62210 Cuernavaca, Mor., Mexico; neri@ibt.unam.mx (E.N.-C.); joserobertoponcelopez21@gmail.com (R.P.-L.); aolvera@ibt.unam.mx (A.O.-R.); alagon@ibt.unam.mx (A.A.); 4 Department of Biology, University of Central Florida, 4000 Central Florida Blvd., Orlando, FL 32816, USA; Jason.Strickland@ucf.edu; 5 Department of Biological Sciences and Department of Forestry and Environmental Conservation, Clemson University, 190 Collings St., Clemson, SC 29634, USA; viper@clemson.edu; 6 Instituto Clodomiro Picado, Facultad de Microbiología, Universidad de Costa Rica, San José 11501, Costa Rica; bruno.lomonte@ucr.ac.cr

**Keywords:** Mojave Rattlesnakes, Mojave toxin, PLA_2_s, SVMPs, venom phenotypes, hemorrhagic activity, *Crotalus scutulatus scutulatus* individuals with Type A, Type A + B, and Type B venoms were confirmed in Mexico. Proteolytic and biological activity shows high variation among individuals with a specific geographic pattern.

## Abstract

Rattlesnake venoms may be classified according to the presence/absence and relative abundance of the neurotoxic phospholipases A${}_{2}$s (PLA${}_{2}$s), such as Mojave toxin, and snake venom metalloproteinases (SVMPs). In Mexico, studies to determine venom variation in Mojave Rattlesnakes (*Crotalus scutulatus scutulatus*) are limited and little is known about the biological and proteolytic activities in this species. Tissue (34) and venom (29) samples were obtained from *C. s. scutulatus* from different locations within their distribution in Mexico. Mojave toxin detection was carried out at the genomic (by PCR) and protein (by ELISA) levels for all tissue and venom samples. Biological activity was tested on representative venoms by measuring LD${}_{50}$ and hemorrhagic activity. To determine the approximate amount of SVMPs, 15 venoms were separated by RP-HPLC and variation in protein profile and proteolytic activity was evaluated by SDS-PAGE (*n* = 28) and Hide Powder Azure proteolytic analysis (*n* = 27). Three types of venom were identified in Mexico which is comparable to the intraspecific venom diversity observed in the Sonoran Desert of Arizona, USA: Venom Type A (∼Type II), with Mojave toxin, highly toxic, lacking hemorrhagic activity, and with scarce proteolytic activity; Type B (∼Type I), without Mojave toxin, less toxic than Type A, highly hemorrhagic and proteolytic; and Type A + B, containing Mojave toxin, as toxic as venom Type A, variable in hemorrhagic activity and with intermediate proteolytic activity. We also detected a positive correlation between SVMP abundance and hemorrhagic and proteolytic activities. Although more sampling is necessary, our results suggest that venoms containing Mojave toxin and venom lacking this toxin are distributed in the northwest and southeast portions of the distribution in Mexico, respectively, while an intergradation in the middle of both zones is present.

## 1. Introduction

Venoms of snakes belonging to the Family Viperidae are comprised of a complex mixture of proteins that can be grouped into families based on their structural similarities [1]. The most common protein families in rattlesnake venoms are: Zn${}^{2+}$ metalloproteinases, phospholipases A${}_{2}$s (PLA${}_{2}$s), serine proteinases, C-type lectins (CTLs), disintegrins, L-amino acid oxidase (LAAOs), cysteine rich secretory proteins (CRiSPs), bradykinin potentiating peptides (BPPs), and myotoxins [2,3,4,5,6,7]. It has been proposed that rattlesnake venoms be classified into two groups according to their toxicity and proteolytic activity: Type I venoms are characterized by high proteolytic activity and moderate toxicity, while Type II venoms are more toxic but their proteolytic activity is lower or non-detectable [2]. The family responsible for most of the proteolytic activity in Type I venom is the snake venom metalloproteinase (SVMPs). In Type II venoms, the protein that is responsible for their high toxicity is a presynaptically acting β-neurotoxic PLA${}_{2}$ called Mojave toxin (MTX) originally described in Mojave Rattlesnakes (*Crotalus scutulatus scutulatus*) but also found in *C. viridis*, *C. oreganus*, *C. mitchelli*, and *C. tigris* [8,9]. This toxin is homologous in structure to Crotoxin in *C. durissus* and *C. vegrandis*, and Canebrake toxin in *C. horridus* [8,10].

SVMPs are enzymes formed by multiple domains whose main toxic effects are due to the disruption of the hemostatic system. SVMPs are classified into three groups (P-I, P-II, and P-III) according to the number of domains they contain. PI SVMPs are composed of only a metalloproteinase domain (M) while P-II SVMPs contain an additional disintegrin domain (D). P-III SVMPs are further divided into P-IIIa, P-IIIb, P-IIIc and P-IIId based on post-translational modifications that these proteins can exhibit including presence of additional domains and domain hydrolysis and dimerization [11]. SVMPs induce several pathophysiological effects such as myonecrosis, blistering, inflammation, coagulopathy, and inhibition of platelet aggregation, but are most known for their capacity to generate local and systemic hemorrhage [12]. Bleeding has been associated with the ability of SVMPs to hydrolyze key components of basal membranes such as type IV, VI and XV collagens and perlecan [13]. Additionally, SVMPs are known for their proteolytic activity on other substrates such as Hide Powder Azure (HPA), casein, azocasein, and some proteins of the coagulation cascade including fibrin, fibrinogen, prothrombin, and factor X [14,15].

Mojave toxin, isolated for the first time from Mojave Rattlesnake venom, is a heterodimeric PLA${}_{2}$ composed of one non-enzymatic acidic subunit (90 amino acids, 9.6 kDa, pI = 3.6) and one enzymatic basic subunit with PLA${}_{2}$ activity (128 amino acids, 14.6 kDa, pI = 9.6) associated by non-covalent bonds [16]. Neurotoxic, cardiotoxic, and myonecrotic activities have been attributed to MTX and its toxicity has been calculated to be as low as 0.056 mg/kg i.v. via LD${}_{50}$ tests in mice [16,17,18,19]. MTX is encoded by two genes composed of four exons and three introns each [20] and it is necessary that both MTX genes be expressed for the completely functional protein to be present in the venom [21]. MTX is structurally similar to other *Crotalus* neurotoxins such as Crotoxin (from the *C. durissus* complex). Both toxins share 96% and 99% of amino acid sequence identity for the acidic and basic subunits, respectively, and induce similar biological effects [22,23,24]. Previous reports have shown that antibodies against the basic subunit of Mojave toxin are able to react with the basic subunit of Crotoxin from *C. d. terrificus* venom, corroborating the high structural similarity between both proteins [25,26].

The dichotomy between toxicity and proteolytic activity can also be found within a single species of rattlesnake. In these cases, the phenotypes are referred to as Type A (neurotoxic and with scarce hemorrhagic and proteolytic activities—similar to Type II) and Type B (hemorrhagic and proteolytic and without neurotoxins—similar to Type I). Intra-specific variation in biochemical and biologic properties of rattlesnake venoms has been broadly documented [27,28,29,30]. Particularly, geographic venom variation has been demonstrated in several rattlesnakes including Mojave Rattlesnakes (*C. scutulatus scutulatus*). Three venom phenotypes were originally described for populations of Mojave Rattlesnakes from the Sonoran Desert in the United States: Type A, Type B, and Type A + B (containing a combination of neurotoxic, hemorrhagic, and proteolytic activities) [31,32,33]. Massey et al. [5] extended this classification to six venom phenotypes (A, B, C, D, E, and F) varying from each other in the presence and abundance of three main proteins including Mojave toxin, SVMPs and myotoxin *a* (herafter myotoxin). The first three phenotypes (A, B, and C) contain mostly SVMPs and very little if any Mojave toxin or myotoxin. The last three phenotypes (D, E, and F) are composed mostly for Mojave toxin and myotoxin with scarce or no SVMPs.

Mojave Rattlesnakes are distributed in the southwestern United States and Mexico [34]. In Mexico, the subspecies *C. s. scutulatus* can be found in the states of Aguascalientes, Chihuahua, Coahuila, Durango, Guanajuato, Jalisco, Nuevo León, Queretaro, San Luis Potosí, Sonora, Tamaulipas, and Zacatecas [34]. Although the composition of Mojave Rattlesnake venom in the United States has been broadly studied, in Mexico the only study of venom composition was carried out in La Comarca Lagunera, Mexico, a region located at the north-central portion of Mexico in the states of Coahuila and Durango. In this region, *C. s. scutulatus* displayed venom devoid of Mojave toxin and with biochemical and biological activities similar to Type B venom from the Sonoran Desert in the United States [35]. However, it has not been determined if Mojave rattlesnake individuals with neurotoxic (Type A) and/or neurotoxic and hemorrhagic (Type A + B) venom are present in Mexico nor whether Type B venom in Mexico and the U.S. are the same. Therefore, the goal of this study was to determine if the venom of Mojave Rattlesnakes in the southern portion of *C. s. scutulatus*’ distribution is as diverse as it is in the U.S.A.

## 2. Results

### 2.1. Mojave Toxin Detection via PCR

Thirty-four *C. s. scutulatus* DNA samples from Mexico were tested for the presence of the genes of the two Mojave toxin subunits and compared to neurotoxic and non-neurotoxic *C. s. scutulatus* controls from Arizona. PCR amplification with both the acidic (MTXA) and basic (MTXB) primers generated fragments of ∼1000 bp in 41% of the samples (Table 1, Table 2 and Table 3).

**Figure 1:**
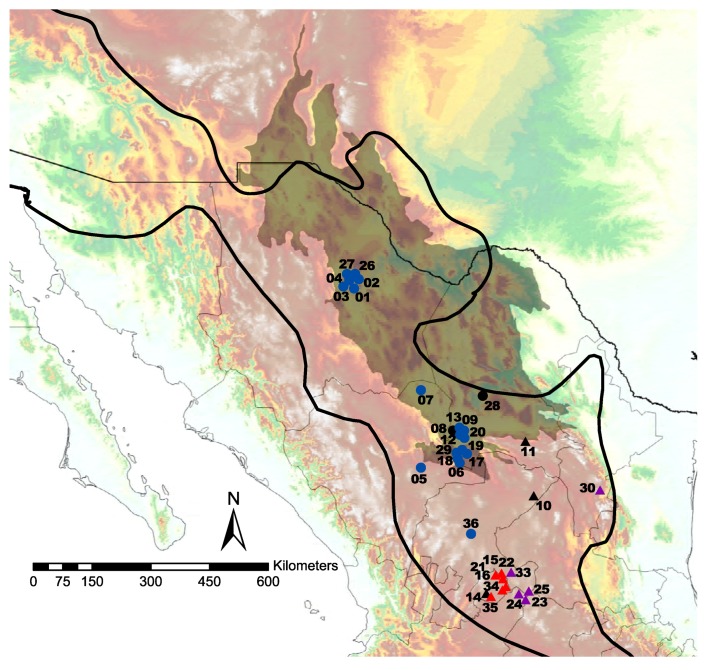
Geographic distribution of *C. s. scutulatus* individuals with and without Mojave toxin genes in Mexico. Individuals containing Mojave toxin are represented with triangles (red = Type A, purple = TypeA + B, and black = collected dead), while individuals lacking Mojave toxin genes (Type B) are shown with circles (blue = venom assays conducted, black = collected dead). Numbers correspond to the individuals in Table 1, Table 2 and Table 3 without the C.S.S. abbreviation. The outline zone represents *C. s. scutulatus*’ distribution, the shaded area represents the Chihuahuan Desert, and colors on the map represent elevation where light blue is closest to sea level and white is highest above sea level.

In general, individuals containing acidic and basic subunits were found in the southeast portion of the sampling area, while snakes lacking Mojave toxin genes were distributed in the northwest region (Figure 1). Five samples (CSS08, CSS10, CSS11, CSS14 and CSS28, from Matamoros, Coahuila; San Tiburcio, Zacatecas; General Cepeda, Coahuila; Aguascalientes, Aguascalientes; and El Mezquite, Coahuila, respectively) were only analyzed for the presence of the Mojave toxin genes because they were collected dead on the road and no venom was available (Figure 1).

Therefore, these five samples were not included in Table 1, Table 2 and Table 3 because these assays were not possible. Samples CSS10, CSS11 and CSS14 were positive for both Mojave toxin genes. To corroborate that fragments obtained by PCR correspond to MTXA, the fragment obtained with the acidic subunit primers from individual CSS11 was sequenced and analyzed for similarities with other sequences on GenBank. Our sequence (GenBank Accession MG574869) is 99% percent similar to the acidic subunit of Mojave toxin from *C. s. scutulatus* from the United States (GenBank Accession KX211993 [36]).

### 2.2. Mojave Toxin Detection by ELISA

Twenty-nine *C. s. scutulatus* venoms were tested for Mojave toxin using ELISA (Table 1, Table 2 and Table 3). Eleven venoms (38%) tested positive for Mojave toxin (Table 2 and Table 3). All individuals containing Mojave toxin in their venom were positive for both Mojave toxin genes using PCR and no individuals containing Mojave toxin genes lacked the protein in their venom. The approximate amount of Mojave toxin in venoms calculated by ELISA was variable, ranging from 12% (CSS16) to 29.5% (CSS23) (Table 2 and Table 3). The percentage of Mojave toxin in the Type A venom control from Arizona was 37.9%. As expected, the Type B venom control did not contain Mojave toxin.

### 2.3. Median Lethal Dose (LD${}_{50}$)

Eighteen venoms were analyzed for toxicity in mice (Table 1, Table 2 and Table 3). LD${}_{50}$ values for Mexican neurotoxic venoms ranged from 0.092 mg/kg to 0.203 mg/kg with an average of 0.143 mg/kg (Table 2 and Table 3). In addition, mice injected with these venoms displayed flaccid paralysis of the hind limbs, a neurotoxic effect. In contrast, venoms lacking Mojave toxin were less toxic with LD${}_{50}$ values range from 0.300 mg/kg to 0.890 mg/kg with an average of 0.660 mg/kg (Table 1). Based on this, venoms containing Mojave toxin were approximately five times more toxic than venoms lacking the neurotoxin. The controls from Arizona had LD${}_{50}$ values of 0.13 mg/kg and 1.09 mg/kg for Type A and Type B venoms, respectively.

### 2.4. Hemorrhagic Activity

Twenty-six venoms were tested for hemorrhagic activity (Table 1, Table 2 and Table 3). Fifteen venoms lacking Mojave toxin induced significant hemorrhagic activity (values of 3 and 4) and were classified as Type B (Table 1). Interestingly, five neurotoxic venoms also caused hemorrhaging although with variable intensity; these venoms were classified as Type A + B (Table 3). Venoms CSS23, CSS24, CSS25 from Ojuelos, Jalisco caused hemorrhage with values of 2, 3, and 2, respectively, while venoms from Genaro Garcia, Zacatecas (CSS22), and La Ascensión, Nuevo León (CSS30) had values of 4. Six neurotoxic venoms from Aguascalientes, and the neurotoxic control did not cause hemorrhaging and were classified as Type A (Table 2). The Type B venom from Arizona had a hemorrhage value of 3 (Table 1).

### 2.5. Hide Powder Azure (HPA) Hydrolysis

Twenty-seven venoms were tested for HPA hydrolysis (Table 1, Table 2 and Table 3). Venoms displayed variable proteolytic activity to HPA. Venoms lacking Mojave toxin (Type B) were the most proteolytic with values ranging from 31.9 to 46.5 U/mg (Table 1). Venoms with both Mojave toxin and hemorrhagic activity (Type A + B) hydrolyzed HPA in values ranging from 21.8 to 39.8 U/mg (Table 3). Finally, neurotoxic venoms without hemorrhagic activity (Type A) were less proteolytic than the first two groups of venoms with values of enzymatic activity from 2.6 to 5.8 U/mg (Table 2).

### 2.6. Reverse Phase HPLC for MTX and SVMPs Detection

**Figure 2:**
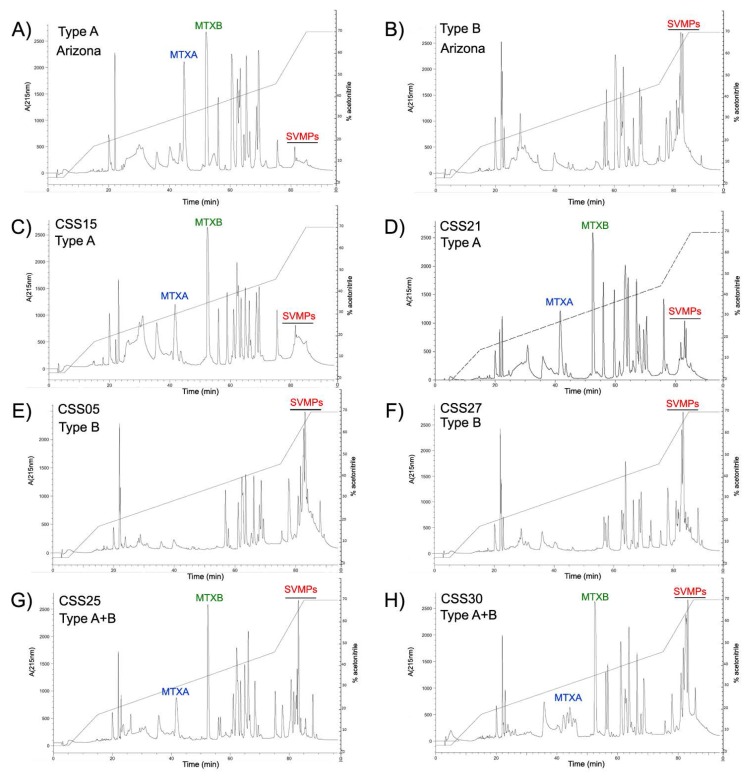
Representative reverse-phase HPLC chromatograms of the three *C. s. scutulatus* venom types found in Mexico. (**A**) Type A control venom from Arizona; (**B**) Type B control venom from Arizona; (**C**,**D**) Type A venoms from Mexico; (**E**,**F**) Type B venoms from Mexico; (**G**,**H**) Type A + B venoms from Mexico. Retention time is along the x axis for each panel and labeled every twenty minutes. Proteins were detected at 215 nm and absorbance is indicated on the left axis. The acetonitrile gradient is shown in the HPLC graph and the percentage value corresponds to the right axis for each panel. Acidic (MTXA) and basic (MTXB) subunits of Mojave toxin and snake venom metalloproteinases (SVMPs) are illustrated in blue, green and red, respectively. The remaining chromatograms are in Figure A1.

**Figure A1:**
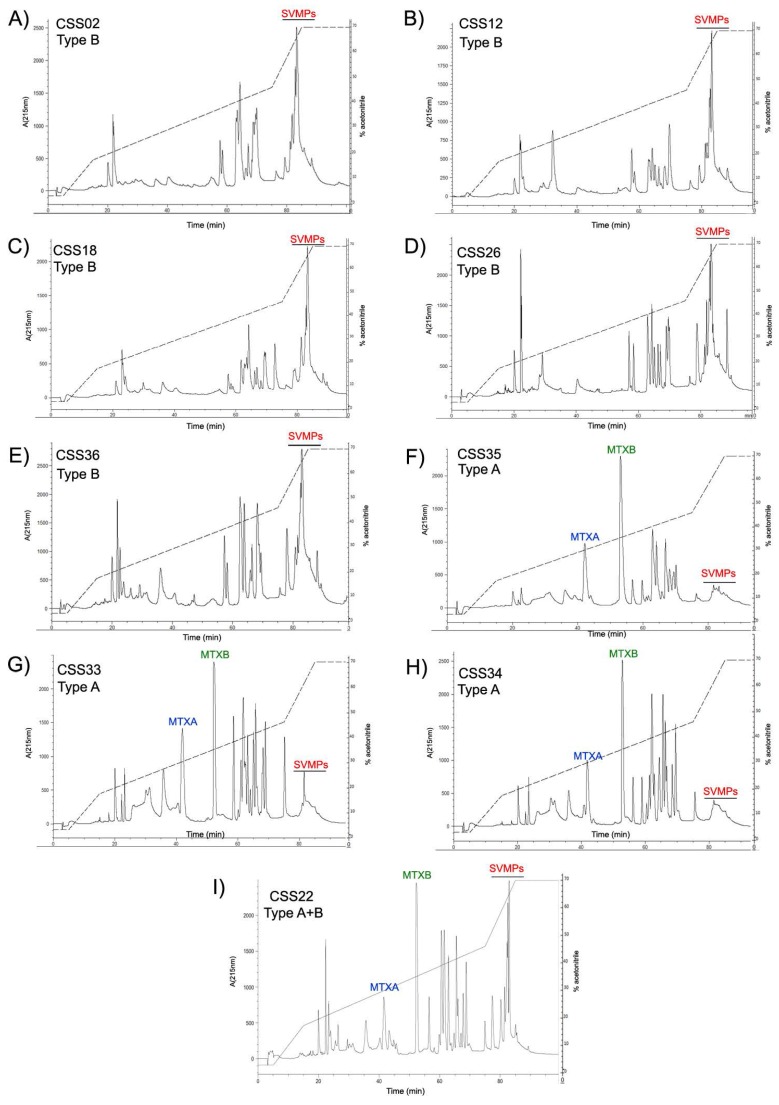
Reverse-phase HPLC chromatograms not shown in Figure 2 of the three *C. s. scutulatus* venom types found in Mexico: (**A**–**E**) Type B venoms from Mexico; (**F**–**H**) Type A venoms from Mexico; and (**I**) Type A + B venom from Mexico. Retention time is along the x axis for each panel and labeled every twenty minutes. Proteins were detected at 215 nm and absorbance is indicated on the left axis. The acetonitrile gradient is shown in the HPLC graph and the percentage value corresponds to the right axis for each panel. Acidic (MTXA) and basic (MTXB) subunits of Mojave toxin and snake venom metalloproteinases (SVMPs) are illustrated in blue, green and red, respectively.

Fifteen representative venoms (Type A—CSS15, CSS21, CSS33, CSS34 and CSS35; Type B—CSS02, CSS05, CSS12, CSS18, CSS26, CSS27 and CSS36; and Type A + B—CSS22, CSS25 and CSS30) from Mexican *C. s. scutulatus* and both controls were fractionated by RP-HPLC (representative chromatograms are shown in Figure 2 and remaining in Figure A1).

**Figure 3:**
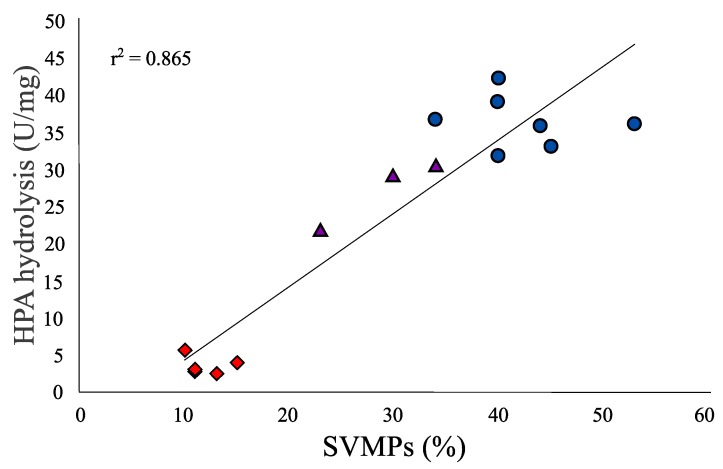
Relationship between the SVMP percentage and proteolytic activity (HPA hydrolysis) of venoms from fifteen *C. s. scutulatus* from Mexico. Type A, B and A + B venoms are red diamonds, blue circles, and purple triangles, respectively.

The fraction that eluted at 42 min was the acidic subunit of Mojave toxin based on our MALDI-TOF-TOF analysis which obtained the three chains that make up the subunit: (1) alpha chain: SSYGCYCYCGAGGAGGQGWPWPQDASDRCCFEHDCCYAKLTGCDPTTTD; (2) beta chain: RQEDGEIVCGGDDPCGTQQICECDKAAAICFRDSMN; and (3) gamma chain: RFSPENCQGESQPC (Figure 2). This matched the sequence reported for the acidic subunit of Mojave toxin in the Uniprot database (P18998) [22]. A high peak eluting at approximately 52 min was detected in Type A and Type A + B venoms but was absent in Type B venoms (Figure 2). This peak corresponded to the basic subunit of Mojave toxin as demonstrated by N-terminal sequence analysis (HLLQFNKMIKFETR) and its molecular weight was 14.2 kDa and 14.3 kDa (by mass spectrometry), indicating the presence of at least two isoforms. The percentage of SVMPs in Type A, Type B, and Type A + B venoms was notably different (Table 1, Table 2 and Table 3 and Figure 2). Type A venom from Arizona and five venoms from Aguascalientes showed lower SVMPs abundance (1–15%) compared to the other eleven venoms analyzed. Conversely, the percentage of SVMPs in non-neurotoxic venoms ranged from 34–53%. Chromatograms of venoms containing neurotoxic and hemorrhagic activities revealed an intermediate SVMPs percentage with values from 23–34%. A significant positive correlation (r${}^{2}$ = 0.865; F = 84.4; df = 1.13; *p* < 0.001) was observed between the SVMPs percentage and proteolytic activity (HPA hydrolysis) of Mexican *C. s. scutulatus* venoms, suggesting that as the amount of SVMPs increases, the proteolytic activity also increased (Figure 3).

### 2.7. SVMPs Detection by Western Blot

**Figure 4:**
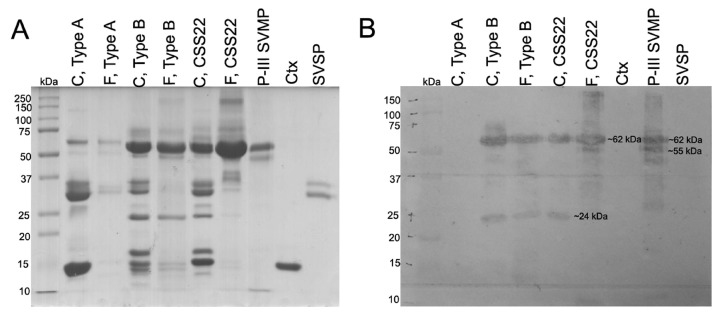
(**A**) SDS-PAGE; and (**B**) Western blot analysis for SVMPs detection of Type A and Type B venoms from Arizona as well as CSS22 venom from Mexico. Whole crude venoms are indicated with C and reverse-phase HPLC fractions eluted after 78 min are indicated with F. Ctx = Crotoxin; SVSP = snake venom serine protease; P-III SVMP = type III snake venom metalloproteinases.

To confirm the fractions eluted after 78 min by RP-HPLC were SVMPs, a Western blot analysis was carried out. Figure 4A shows a SDS-PAGE with the venoms and fractions that were transferred to nitrocellulose membrane. Antibodies against SVMPs were unable to bind SVMPs in Type A venom (Figure 4B) or its fractions (data not shown).

Two antibody recognition zones (∼24 kDa and ∼62 kDa) were detected in whole Type B and CSS22 venoms and the reverse-phase HPLC fractions from the Type B venom. Unexpectedly, reverse-phase HPLC fractions from the CSS22 venom lacked the ∼24 kDa band but displayed an additional band of ∼55 kDa similar to the P-III SVMP used as positive control. No bands were seen in the lanes corresponding to Crotoxin and SVSPs which corroborates the antibody’s specificity.

### 2.8. SDS-PAGE

**Figure 5:**
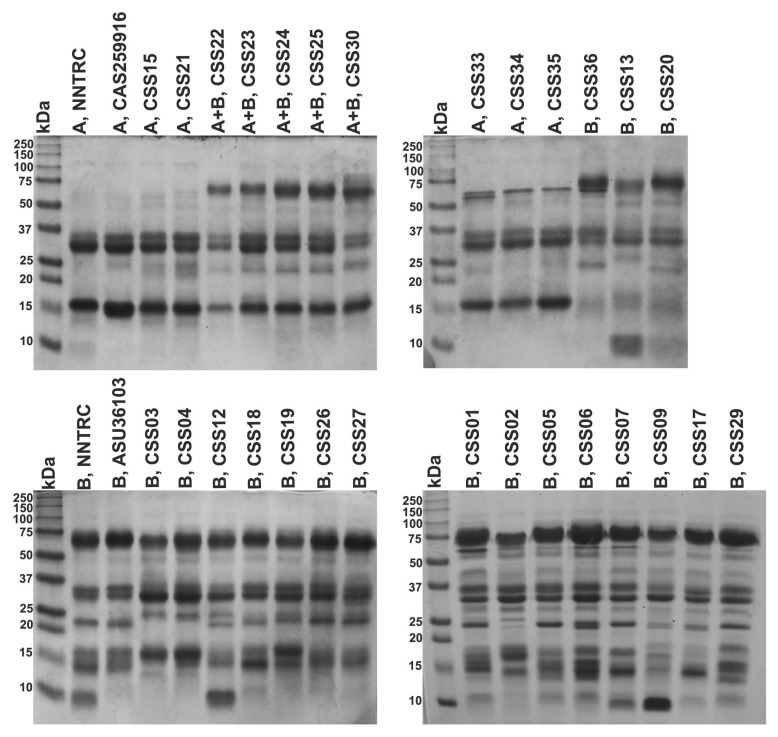
SDS-PAGE of *C. s. scutulatus* venoms from Mexico under reducing conditions. NNTRC A and B correspond to pooled Type A and Type B venoms from National Natural Toxins Research Center, respectively; A, CAS259916 and B, ASU36103 represent individual Type A and Type B venoms from Arizona, respectively. kDa: kilodaltons. Bands with molecular weight in the ranges of 75 to 50 kDa are likely SVMPs and ∼15 kDa are likely PLA${}_{2}$s.

Twenty-eight *C. s. scutulatus* venoms from Mexico and four samples from the USA (two Type A and two Type B) were separated by SDS-PAGE. Notable differences were detected in the presence and intensity of several protein bands among the different venoms analyzed (Figure 5).

The most prominent differences were observed in the range of 50 to 75 kDa, where venoms displayed a prominent band of 62 kDa (likely PIII-SVMPs); however, this band was much less visible in the five Type A venoms from Aguascalientes (CSS15, CSS21, CSS33, CSS34, and CSS35). Inversely, venoms with faint 62 kDa band displayed an intense band of 15 kDa (likely the basic subunit of Mojave toxin). The zone near 15 kDa was more heterogeneous (in number and intensity of bands) in venoms lacking Mojave toxin but, in general, bands were less intense than those displayed by venoms with Mojave toxin. Interestingly, ten of the eighteen Type B Mexican venoms (CSS01, CSS05, CSS06, CSS07, CSS09, CSS12, CSS13, CSS17, CSS20, and CSS29) and the Type B control from NNTRC (National Natural Toxins Research Center in Kingsville, Texas) had a small band of less than 10 kDa, which was absent in the rest of venoms and likely corresponds to myotoxins.

## 3. Discussion

We found Mojave toxin in 11 of 29 individuals of *C. s. scutulatus* from Mexico at both the genomic (PCR) and protein (ELISA) levels. Additionally, three samples from Mojave Rattlesnakes collected dead were also positive for Mojave toxin using PCR (CSS10, CSS11, and CSS14), although we could not confirm the presence of Mojave toxin in their venom because venom could not be collected. However, considering that in all other samples where Mojave toxin genes were amplified, the protein was observed in the venom, it is highly probable that these three individuals had Mojave toxin in their venom as well. We successfully determined the presence of Mojave toxin in *C. s. scutulatus* venoms from Mexico using monoclonal and polyclonal antibodies against Crotoxin. We did not find any individuals that were negative for Mojave toxin by PCR and positive by ELISA, indicating the specificity of the antibodies for neurotoxic PLA${}_{2}$s (Table 1, Table 2 and Table 3). These results corroborate the effectiveness of both techniques to detect Mojave toxin in *C. s. scutulatus* and are in agreement with other reports [21,37,38]. In addition, we confirmed the presence of Mojave toxin via RP-HPLC. Venoms that were positive for Mojave toxin in our ELISA anaylsis show two peaks at approximately 42 and 52 min in our chromatograms that were absent in venoms negative for Mojave toxin based on ELISA (Figure 2). We confirmed, via mass spectrometry and N-terminal sequencing analysis, that these two peaks correspond to the acidic and basic subunites of Mojave toxin, respectively. Using the same gradient, Castro et al. [27] detected the basic subunit of crotoxin at the same retention time in venoms of *C. simus* from Mexico. This indicates similar physiochemical characteristics between both neurotoxins.

We observed and characterized three types of venoms within *C. s. scutulatus* in Mexico according to their biological and biochemical activities and the presence/absence of Mojave toxin. First, Type A, containing Mojave toxin, was highly toxic, lacked hemorrhagic activity, and its proteolytic activity was minimal (Table 2). Second, Type A + B, expressed Mojave toxin, was as toxic as the Type A venom, was hemorrhagic, and its capacity to hydrolyze HPA was intermediate (Table 3). The third, Type B, did not contain Mojave toxin, its LD${}_{50}$ was several times higher (i.e. less toxic) than the first two venom types, and was more hemorrhagic and proteolytic than the other two venom types (Table 1). This demonstrates conclusively that the three phenotypes are not just found in Arizona [39] and that venom variability of *C. s. scutulatus* in Mexico is just as diverse as it is in southern Arizona [5].

In other studies, it has been demonstrated that SVMPs are the proteins that elute last in RP-HPLC [5,27,40]. Similar to these reports, we detected important variation in the number and height of the peaks in the area after 78 min of elution in fifteen *C. s. scutulatus* venoms from Mexico (Figure 2). We confirmed via Western blot that this area contained SVMPs (Figure 4). Although only three venoms (Type A and Type B controls, and a venom Type A + B from Mexico) were tested for the presence of SVMPs by Western blot, it is clear that the amount of SVMPs in venom Type A is minimal or absent completely (no bands were detected by Western blot), while Type B and A + B venoms contained at least two groups of SVMPs according to their molecular mass: ∼55 kDa and ∼62 kDa likely P-III isoforms and ∼24 kDa likely P-I SVMPs (Figure 4). These results were congruent with those observed in SDS-PAGE where venoms without hemorrhagic activity displayed thin bands in the zone of 50–75 kDa indicating minimal presence of P-III SVMPs (Figure 5). Our results also agree with those of Massey et al. [5] which reported that Mojave Rattlesnakes with Type B or Type A + B venom from Arizona contain two types of SVMPs: P-I (23 kDa) and P-III (48 kDa and 69 kDa), with the P-III being more abundant.

A positive correlation between the amount of SVMPs and proteolytic and hemorrhagic activities was noted (Figure 3). Venoms with the highest amount of SVMPs were the most proteolytic and hemorrhagic (Table 1). Inversely, venoms with a limited amount of SVMPs were least proteolytic and hemorrhagic. Dagda et al. [41] proposed for *C. s. scutulatus* that variation in hemorrhagic and proteolytic activities can be associated with structural differences in the metalloproteinase domain and/or differential metalloproteinase gene expression ligated to polymorphisms in the gene region that encode the metalloproteinase domain. In agreement with this hypothesis, variation in the proteolytic (gelatinase) activity related to SVMPs expression variation has been reported in the Eastern Diamondback Rattlesnake (*C. adamanteus*) indicating that differential gene expression can have an important influence in the venom’s biological activities [42].

The variation in biological and biochemical activities in the venom of individual *C. s. scutulatus* specimens from Mexico reported here could have important implications in human envenomation presented in this country. In Mojave Rattlesnakes from the United States, neurotoxic venoms were approximately seven times more toxic in mice than the hemorrhagic ones [33]. Similarly, neurotoxic venoms from Mexico were about five times more lethal than venoms lacking Mojave toxin. The probability of intubation or death increases with the presence of neurotoxic components in venom [5]. Thus, despite the lack of clinical reports that would help to have a better correlation between venom types and clinical effects, more severe envenomation could be expected in areas of Mexico where Type A or Type A + B venoms are distributed. Moreover, regional variation in the number and relative abundance of the protein families in snake venoms has important implications in the antivenom effectiveness. Due to antivenoms being made with different venom immunization mixtures, only those proteins with similar structure to those used in the immunization mixture will be recognized by the antivenom. Hence, if venoms in some areas are quite different to those used in the immunization of animals for the antivenom production, the capacity of antivenom to neutralize these particular toxins will be limited [43]. For example, the Mexican antivenom Antivipmyn had difficulties neutralizing neurotoxic venoms of *C. s. scutulatus* from Arizona and Texas and *C. s. salvini* from Mexico [44]. Therefore, knowledge of the areas in Mexico with variable venom is important to improve the selection of the venom mixture used to create Mexican antivenom.

Several hypotheses have been proposed to explain geographic venom variation in rattlesnakes. These hypotheses include rattlesnake hybridization [21,30], prey size (the bigger the prey, more metalloproteinases required to digest), geographic latitude and/or altitude (if the environmental thermal conditions are quite variable such as extremes of latitude and/or altitude, more metalloproteinases are required to degrade tissues) [2], and prey stability in the habitat (places where a substantial recent shift in prey has occurred, Mojave toxin is retained as result of strong selection for high toxicity) [45]. In Mexico, *C. s. scutulatus* are predominately distributed in two physiographic regions: uplands and lowlands of the North and the Central Mesa. The uplands and lowlands of the North are relatively flat and located in three states, Chihuahua, Coahuila, and Durango, with an arid/semiarid climate and an annual amount of precipitation varying from 125 to 400 mm. The average altitude is approximately 1000 m.a.s.l. The vegetation in this area is comprised primarily of desert microphyllous matorral and desert rosetephylous matorral [46]. On the other hand, the Central Mesa includes portions of the states of Zacatecas, San Luis Potosi, Guanajuato, Durango, Jalisco, Aguascalientes, and Queretaro. The minimum elevation in the Central Mesa province is 1180 m.a.s.l, while the highest point is 3200 m.a.s.l. The climate in this area is semiarid; however, differences in elevation results in sharp contrast between close localities in their climatic conditions. The precipitation ranges from 300 mm to 900 mm. The vegetation consists of rosetophyllous matorrals and matorral with cacti and natural grasslands. An interesting feature of the climate in this area is its inverted zonality: the temperatures decrease and precipitation increases from north to south [46]. Individuals with venom Type A and Type A + B were found mainly in the Central Mesa (a higher and cooler place), while individuals with venom Type B were located mostly in the uplands and lowlands of the North (lands with low altitudes and higher temperatures, Figure 1). The only exception was the individual CSS36 from Plateros, Zacatecas who lacked Mojave toxin despite having been found at higher elevation comparing to the other Type B venoms. A similar pattern was observed in *Crotalus oreganus helleri* venom where geographic venom variation was related to altitude differences, so, individuals living in the higher altitude and greatest temperature fluctuations had Type A venom lacking SVMPs almost entirely [29]. Although more sampling is needed, it is possible that climatic differences in the subregions (that might represent different evolutionary environmental pressures) are, in part, associated with the venom variation seen in the *C. s. scutulatus* from Mexico. This hypothesis would need to be tested in conjunction with environmental data. In addition, other studies have hypothesized that transition between lowland Chihuahuan desert habitat and higher elevation semi-arid habitat in the Central Mesa could have formed a biogeographic barrier for some species [47,48]. Finally, considering that Mojave Rattlesnakes with venom Type A + B were found in an area located in the middle between individuals with venom B (located further north) and A (located further south), an intergrade zone is likely, similar to the one described in Arizona [32].

## 4. Conclusions

In conclusion, *C. s. scutulatus* individuals with Type A, Type A + B, and Type B venoms are distributed in Mexico. Individuals with Type B venom seem to be distributed in the northwest region and individuals with Type A and A + B venoms are found in the southeast region. Additionally, we found an interesting tendency in which individuals containing Mojave toxin were generally detected in areas with higher elevation compared to Type B venoms, however, this same tendency has not been noted in *C. s. scutulatus* individuals from the United States. In this manner, more sampling is needed to completely delimit the areas where *C. s. scutulatus* with venoms A, B, and A + B are distributed in Mexico and to test the hypothesis that altitude is an important environmental factor influencing the venom variation in the Mojave rattlesnake.

As previously reported, venoms lacking Mojave toxin were more hemorrhagic and proteolytic than venoms containing the neurotoxin and this difference is possibly associated to variations in the amount of SVMPs present in the venoms. Mexican Mojave Rattlesnakes with Type A and Type B venom were similar in biological (toxicity and hemorrhagic activity) and biochemical (proteolytic activity and protein profile) properties to those from Arizona used as controls and published studies of *C. s. scutulatus* in the U.S.

## 5. Materials and Methods

### 5.1. Ethics Statement

We followed the guidelines described by the live animal use committees of the Facultad de Ciencias Biológicas at UJED, the Universidad Nacional Autónoma de México, and the Secretaría de Medio Ambiente y Recursos Naturales (SEMARNAT) of Mexico as well as developing our protocols in accordance with the American Society of Ichthyologists and Herpetologists guidelines for use of live amphibians and reptiles. Animal care and use protocols involving snakes in the USA were approved by UCF’s Institutional Animal Care and Use Committee under protocol 13-17W. SEMARNAT issued collecting permits (SGPA/DGVS/01090/17 and 03562/15) for samples collected in Mexico and the controls from the U.S. were collected under State of Arizona Game and Fish Department (SP628489, SP673390, SP673626, SP715023).

### 5.2. *Crotalus scutulatus scutulatus* Sampling

Blood and venom samples were obtained from twenty-nine *C. s. scutulatus* from different areas of Mexico. Additionally, five tissue samples of dead individuals were collected from animals that were hit by cars. Venom and blood from two Mojave Rattlesnakes with Type A and Type B venom from Arizona were used as controls. To reduce the number of mice sacrificed in this study and to conserve venom (snakes were milked once and a variable amount of venom was obtained from each snake), venom analyses were prioritized based on importance and amount of venom available for subsequent analyses.

### 5.3. PCR Detection of Mojave Toxin Genes

Genomic DNA was extracted from blood or tissue using the DNeasy^®^ Blood and Tissue kit (QIAGEN, Valencia, CA, USA) following the manufacturer’s instruction. Detection of portions of the MTXA (F 5${}^{\prime }$-TGCGGGGAGAAGTGGTATTT-3${}^{\prime }$; R 5${}^{\prime }$-GCAATTTTCGGGCGAGAACC-3${}^{\prime }$) and MTXB (F 5${}^{\prime }$-ACCTGCTGCAATTCAACAAGA-3${}^{\prime }$; R 5${}^{\prime }$-CGAGAGTCCGGGTAAAACAT-3${}^{\prime }$) genes was carried out by PCR using the primers designed by Zancolli et al. [21]. The PCR mix was made as follows: DNA (100–300 ng), primers (0.2 μM), Go Taq^®^ DNA polymerase

(Promega, Fitchburg, WI, USA, 0.5 units), dNTP mix (0.1 mM), and MgCl${}_{2}$ (1.5 mM), in a total volume of 10 μL. PCR was conducted starting with a heat denaturation step at 94 ${}^{\circ }$C for 5 min, followed by 35 cycles of heat denaturation at 90 ${}^{\circ }$C for 30 s, annealing for 60 s, and extension at 72 ${}^{\circ }$C for 1.5 min, and a final extension at 72 ${}^{\circ }$C for 5 min. The PCR annealing temperatures for MTXA and MTXB were 61 ${}^{\circ }$C and 62.8 ${}^{\circ }$C, respectively. Fragments were visualized with Gel Red in a 1% agarose gel after electrophoresis. Additionally, the PCR product generated with the primers for acidic subunit from individual CSS11 was further purified and sequenced at the Massive DNA Sequence Unit, Instituto de Biotecnología, UNAM to corroborate that the fragment amplified corresponded to MTXA.

### 5.4. Protein Concentration Determination

The protein concentration in the venoms was determined in triplicate using the Pierce^®^ Bicinchoninic Acid (BCA) Protein Assay (Thermo Scientific, Rockford, IL, USA) using bovine serum albumin (BSA) as a standard, according to the manufacturer’s instruction. All analyses that used venom used this to control for the protein concentration.

### 5.5. Mojave Toxin Detection by Sandwich ELISA

ELISA plates (Nunc MaxiSorp) were sensitized for 1 h at 37 ${}^{\circ }$C with 100 μL per well of the monoclonal antibody 4F6 against the basic subunit of Crotoxin to a final concentration of 5 μg/mL in sensitization solution (100 mM NaHCO${}_{3}$, pH 9.5). Then, plates were washed twice with 200 μL of wash solution (50 mM Tris/HCl, pH 8 + 150 mM NaCl + 0.05% Tween 20); this step was repeated after every ELISA step. Plates were blocked with blocking buffer (50 mM Tris/HCl, pH 8 + 0.5% gelatin + 0.2% Tween 20) for 2 h at 37 ${}^{\circ }$C. A standard curve with purified basic subunit of Crotoxin was prepared to 2 μg/mL by making serial dilutions (1:3). In addition, 150 μL of serial dilutions (1:3) of each venom (20 μg/mL) was added per well and incubated for 1 h at 37 ${}^{\circ }$C. One hundred microliters of rabbit polyclonal antibodies against Crotoxin (1 μg/mL) were added per well and incubated 1 h at 37 ${}^{\circ }$C. Rabbit anti-IgG antibodies conjugated to horseradish peroxidase (HRP) diluted 1:4000 were added and incubated for 1 h at 37 ${}^{\circ }$C. Finally, plates were revealed by adding 100 μL of revealing buffer (containing 2,2${}^{\prime }$-azino-bis(3-ethylbenzothiazoline-6-sulphonic acid)). Absorbance was read at 405 nm using a spectrophotometer (Magallan®).

### 5.6. Median Lethal Dose (LD${}_{50}$)

LD${}_{50}$ analysis was carried out as described in Borja et al. [35]. Freeze-dried venoms were redissolved in phosphate buffer solution (PBS), pH 7.2. The lethal dose was determined by injecting different quantities of venom diluted in a total volume of 0.5 mL into the caudal vein of male and female ICR- CD1 mice (18 to 20 g) in groups of three. The percentage of dead mice 24 h after inoculation was plotted against the logarithm of the quantity of venom injected and analyzed with nonparametric methods using the program GraphPad Prism V4.0b.

### 5.7. Hemorrhagic Activity

Hemorrhagic activity was assessed using a modification of the technique described by Dagda et al. [41]. Briefly, 15 μg of each venom dissolved in a total volume of 50 μL PBS was injected subcutaneously in the shaved backs of five mice including both males and females from the ICR-CD1 strain (from 28–30 g). After 3 h, the mice were sacrificed by CO${}_{2}$ inhalation and the dorsal skin was removed and extended over a glass plate. Afterward, the halo of hemorrhage produced by each venom was measured to obtain the average hemorrhagic halo generated. Hemorrhagic activity is reported on a relative scale from 0 to 4, where 4 indicates high hemorrhagic activity (hemorrhagic are greater than 20 mm in diameter) and 0 indicates no hemorrhagic activity [41].

### 5.8. Hide Powder Azure (HPA) Hydrolysis

Hydrolysis of hide powder azure was determined by adding 100 μg of venom to 1 mL of a solution containing 5 mg of HPA in 0.1 M Tris-HCl, pH 8.0. After 2 h of incubation, the reaction was stopped by centrifugation at 14,000 rpm for 5 min. Optical absorbance of the supernatant was determined at 595 nm. Each sample was tested in triplicate. A standard curve of HPA hydrolysis was done adding 50 μL of trypsin (2 mg/mL) to three concentrations of HPA (2, 4 and 6 mg/mL). Venom enzymatic activity was calculated using the standard curve. A unit of enzymatic activity (U) was defined as the amount of venom necessary to digest 1 mg of HPA in a 2 h period at room temperature and reported as specific activity (U/mg) [49].

### 5.9. Reverse Phase HPLC for MTX and SVMPs Detection

Venom proteins were separated by reverse phase HPLC on an analytic C18 reverse-phase column (Vydac^®^, Deerfield, IL, USA, 218 TP 4.6 mm × 250 mm) using an Agilent 1100 chromatograph. In brief, each venom (1 mg) was dissolved in 1.7 mL of water containing 0.1% trifluoroacetic acid (TFA). Elution was performed as described by Neri-Castro et al. [27] at 1 mL/min by applying a gradient toward solution B (acetonitrile, containing 0.1% TFA), as follows: 0% B for 5 min, 0 to 15% B over 10 min, 15 to 45% B over 60 min, 45 to 70% B over 10 min, and 70% B for 9 min. Proteins were detected at 215 nm. For one sample (CSS33), we collected and dried the fractions that eluted at approximately 42 and 52 min and for another sample (CSS22) we collected and dried the effluent collected after 78 min. The fraction that eluted at 42 min likely corresponds to the acidic subunit of Mojave toxin. To verify this, the protein sequence was obtained by tandem mass spectrometry of peptides after digestion of the DTT-reduced and iodoacetamide-alkylated protein with trypsin and analyzed by MALDI-TOF-TOF mass spectrometry on a Proteomic Analyzer 4800-Plus instrument (Applied Biosystems) at the Instituto Clodomiro Picado in Costa Rica. The fraction eluted at 52 min was analyzed by Mass Spectrometry (LCQ Fleet Ion Trap Mass Spectrometer) and N-terminal sequence analysis (PPSQ-31A Protein Sequencer) to verify MTXB presence and fractions eluted after 78 min were used in Western blot analysis using antibodies against SVMPs to verify SVMP presence. The relative abundance (% of the total venom proteins) of SVMPs in seventeen venoms (including controls) was calculated based on the relation of the sum of the areas of the reverse-phase chromatographic peaks eluted after 78 min, which is the time SVMPs begin to elute [27]. Relationship between the SVMPs percentage in venoms and proteolytic activity (HPA hydrolysis) was analyzed using linear regression. Significant was determined at the level of *p* < 0.05.

### 5.10. SVMP Detection by Western Blot

Reverse-phase HPLC fractions eluted after 78 min and whole crude Type A, Type B and CSS22 (as Type A + B Mexican venom control) venoms (15 μg) were separated using SDS-PAGE under reducing conditions (in the presence of β-mercaptoethanol). After electrophoresis, proteins were transferred to a nitrocellulose membrane using a model HEP-1 semi-dry immunotransference chamber (Thermo Scientific). After transference at 400 mA for 1 h, the membrane was blocked with 5% non-fat dry milk diluted in TBST buffer (0.01 M Tris-HCl + 0.15M NaCl + 0.05% of Tween-20, pH 7.5) for 2 h. The membrane was then rinsed three times with TBST and incubated by shaking gently for 1 h at ∼28 ${}^{\circ }$C (room temperature) with rabbit antibodies against P-III SVMPs from *C. simus* in a concentration of 1 μg/mL diluted in 10 mL TBST. After three washes with TBST, the membrane was incubated at room temperature for 1 h with goat antibodies anti-rabbit IgG conjugated to horseradish peroxidase (Thermo Scientific). The membrane was again rinsed with TBST and then developed by adding 1-Step Ultra TMB substrate (Thermo Scientific, Rockford, IL, USA). Purified P-III SVMPs, SVSPs, and Crotoxin were used as controls.

### 5.11. SDS-PAGE

The discontinuous system was used on a Miniprotean III system (BioRad). Venom samples (15 μg) were dissolved in sample buffer (50 mM Tris- HCl, pH 6.8, 25% SDS, 10% glycerol, and 0.002% bromo- phenol blue), in the presence of 5% β-mercaptoethanol. Samples were boiled for 5 min and run on a 15% acrylamide gel. Gels were stained with 0.2% Coomassie brilliant blue R-250, 10% acetic acid, and 25% methanol for 1 h and rinsed in 10% acetic acid and 10% methanol [35]. Standard molecular mass markers (Bio-Rad) were used as references. To compare electrophoretic profile between pooled and individual Type A and Type B venoms from the United States, an additional set of pooled Type A and Type B venoms from The National Natural Toxins Research Center were separated by SDS-PAGE too.

## Figures and Tables

**Table 1:** Biologic and proteolytic activities of *C. s. scutulatus* with Type B venom from Mexico.

ID	Geographic Location	Genomic Mojave Toxin Presence (MTXA/MTXB)	% of MTX in Venom	% of SVMPs in Venom (RP-HPLC)	LD${}_{50}$ (mg/kg)	Hemorrhagic Activity (Intensity)	HPA Hydrolysis (U/mg)
CSS01	Aldama-Coyame, Chih.	No/No	0	ND	ND	3	42.4 ± 1.8
CSS02	Aldama-Coyame, Chih.	No/No	0	40	ND	4	39.3 ± 1.8
CSS03	Aldama-Coyame, Chih.	No/No	0	ND	ND	4	46.5 ± 10.7
CSS04	Aldama-Coyame, Chih.	No/No	0	ND	0.622	4	46.5 ± 3.1
CSS26	Aldama-Coyame, Chih.	No/No	0	40	0.842	3	31.9 ± 0.6
CSS27	Aldama-Coyame, Chih.	No/No	0	45	0.863	3	33.3 ± 2.4
CSS09	Matamoros, Coah.	No/No	0	ND	ND	4	42.4 ± 1.8
CSS12	Matamoros, Coah.	No/No	0	40	0.590	4	42.4 ± 1.8
CSS13	Matamoros, Coah.	No/No	0	ND	ND	ND	ND
CSS20	Matamoros, Coah.	No/No	0	ND	0.566	3	35.6 ± 1.9
CSS06	Tanque Aguilereño, Coah.	No/No	0	ND	ND	ND	35.5 ± 1.1
CSS17	Tanque Aguilereño, Coah.	No/No	0	ND	ND	ND	ND
CSS18	Tanque Aguilereño, Coah.	No/No	0	44	0.300	4	36.0 ± 0.3
CSS19	Tanque Aguilereño, Coah.	No/No	0	ND	0.584	3	32.1 ± 1.8
CSS29	Tanque Aguilereño, Coah.	No/No	0	ND	ND	4	37.2 ± 3.1
CSS05	Peñón Blanco, Durango	No/No	0	53	0.684	4	36.3 ± 0.6
CSS07	Mapimí, Durango	No/No	0	ND	ND	4	42.1 ± 4.2
CSS36	Plateros, Zacatecas	No/No	0	34	0.890	4	36.9 ± 0.9
ASU36103	Arizona, USA	No/No	0	34	1.090	3	34.9 ± 0.2

ND: not determined.

**Table 2:** Biologic and proteolytic activities of *C. s. scutulatus* with Type A venom from Mexico.

ID	Geographic Location	Genomic Mojave Toxin Presence (MTXA/MTXB)	% of MTX in Venom	% of SVMPs in Venom (RP-HPLC)	LD${}_{50}$ (mg/kg)	Hemorrhagic Activity (Intensity)	HPA Hydrolysis (U/mg)
CSS15	Tepezala, Ags.	Yes/Yes	18	15	0.152	0	4.0 ± 0.2
CSS16	Tepezala, Ags.	Yes/Yes	12	ND	0.102	0	5.0 ± 0.3
CSS21	Tepezala, Ags.	Yes/Yes	21.5	13	0.150	0	2.6 ± 0.3
CSS33	El Llano, Ags.	Yes/Yes	27.7	11	0.092	0	2.7 ± 0.3
CSS34	El Llano, Ags.	Yes/Yes	25.6	10	0.177	0	5.8 ± 0.2
CSS35	Rio San Pedro, Ags.	Yes/Yes	22.5	11	0.098	0	3.0 ± 0.4
CAS259916	Arizona, USA	Yes/Yes	37.9	1	0.130	0	5.7 ± 0.7

ND: not determined.

**Table 3:** Biologic and proteolytic activities of *C. s. scutulatus* with Type A + B venom from Mexico.

ID	Geographic Location	Genomic Mojave Toxin Presence (MTXA/MTXB)	% of MTX in Venom	% of SVMPs in Venom (RP-HPLC)	LD${}_{50}$ (mg/kg)	Hemorrhagic Activity (Intensity)	HPA Hydrolysis (U/mg)
CSS22	Genaro García, Zacatecas	Yes/Yes	17	30	0.136	4	29.2 ± 2.5
CSS23	Ojuelos, Jalisco	Yes/Yes	29.5	ND	ND	2	25.5 ± 1.3
CSS24	Ojuelos, Jalisco	Yes/Yes	29	ND	ND	3	39.8 ± 1.3
CSS25	Ojuelos, Jalisco	Yes/Yes	21	23	0.203	2	21.8 ± 0.2
CSS30	La Asención, Nuevo León	Yes/Yes	21.7	34	0.179	4	30.5 ± 2.3

ND: not determined.

## Abbreviations

The following abbreviations are used in this manuscript:

Ags.	Aguascalientes
Chih.	Chihuahua
Coah.	Coahuila
CSS	*Crotalus scutulatus scutulatus*
ELISA	Enzyme-linked Immunosorbent Assay
HPA	Hide Powder Azure
LD${}_{50}$	Median Lethal Dose 50
MDPI	Multidisciplinary Digital Publishing Institute
MTX	Mojave toxin
MTXA	Acidic subunit of Mojave toxin
MTXB	Basic subunit of Mojave toxin
ND	No determined due to lack of venom available
NNTRC	National Natural Toxins Research Center
PCR	Polymerase Chain Reaction
PLA${}_{2}$	Phospholipase A${}_{2}$
RP-HPLC	Reverse-Phased High Performance Liquid Chromatography
SDS-PAGE	Sodium Dodecyl Sulfate Polyacrylamide Gel Electrophoresis
SVMP	Snake Venom Metalloproteinases
SVSP	Snake Venom Serine Protease
UCF	University of Central Florida
UJED	Universidad Juárez del Estado de Durango
UNAM	Universidad Nacional Autónoma de México
